# Wetting Properties of Defective Graphene Oxide: A Molecular Simulation Study

**DOI:** 10.3390/molecules23061439

**Published:** 2018-06-13

**Authors:** Ke Xu, Jicheng Zhang, Xiaoli Hao, Chunbo Zhang, Ning Wei, Chao Zhang

**Affiliations:** 1 College of Water Resources and Architectural Engineering, Northwest A&F University, Yangling 712100, China; twtdq@nwafu.edu.cn (K.X.); zhangjicheng0418@163.com (J.Z.); haoxiaoli19920909@nwafu.edu.cn (X.H.); zhangchunbo@nwafu.edu.cn (C.Z.); 2 Institute of Structural Mechanics, Bauhaus-University Weimar, 99423 Weimar, Germany

**Keywords:** wetting property, graphene oxide, molecular dynamics

## Abstract

In the present work, the wettability of defective graphene oxide (GO) film is studied by molecular dynamics simulations. A water droplet is deposited on the surface of a graphene oxide membrane, and the contact angle is measured by fitting the liquid–vapor interface. Although pristine graphene has few hydrophobic properties with a contact angle of 95°, graphene oxide presents more hydrophilic properties, due to the stronger hydrogen bonds interactions at the interface. Moreover, the introduction of vacancy defects at the graphene oxide surface decreases the wettability of graphene oxide. We find that the contact angle of graphene oxide increases from 70° to 82°, with a defective concentration from 0% to 10%. Our results will help provide a new method for controlling the wetting properties of GO and its additional capabilities in device design for applications.

## 1. Introduction

Graphene oxide (GO) [1,2,3,4,5], a functionalized graphene derivative, has recently attracted much attention for its potential usage in membrane science, due to its advantages of being facile and allowing large-scale production in solution [6,7,8,9]. Because of the controllable chemical functionalization [10] and microstructures of GO, GO will provide an unexpected potential for rational material design and optimization. For example, the concentration of oxygen-containing groups in GO can be tuned [11,12], and the microstructures(e.g., defect) can be optimized through chemical, thermal, or electrochemical methods, which enable GO to have a vast number of applications in functional materials [13,14], coatings [15], and separation membranes [16,17,18,19]. What is more, Walton et al. reported that the motion of drops of water could be induced by producing a gradient of oxygen functional groups on GO, and the wetting property of GO could also be controlled due to the change of oxygen functional groups [10]. Such tunability in surface chemistry provides additional capabilities with regards to device design, for applications ranging from microfluidics to chemical sensing.

Wetting behavior is one of the important properties of GO in the aforementioned applications. GO under typical treatment displays hydrophilic properties, with *θ*_C,GO_ ≈ 30−60° [17,20,21], which thus plays an important role in modulating the structure and properties of GO, and as a result, its applications. Previous studies have focused on the effects of chemical functionalization on the wetting properties of GO. For example, Qi et al. reported that the hydrophilic groups can influence the contact angle of pristine GO [21]. Wei et al. studied the relationship between the wetting properties of GO sheets and oxide group concentration density [12]. They found that the wetting properties enhanced with increasing oxide group concentration density, and the tendency of the wetting contact angle and oxide group concentration density can be well-fitted by the Cassie–Baxter model. However, the authors only considered the defect-free situation in their studies while neglecting defective situation effects, which are inevitable in the process of manufacturing and chemically functionalizing graphite [22,23]. According to previous reports, vacancy defects in the surface can effectively reduce the interfacial interactions and weaken the wetting properties. Li et al. reported wetting and interfacial properties of water on the defective graphene [24]. They found that the droplet contact angle is sensitive to a single-vacancy defect. The contact angle increases from 92° to 99° when only 2% vacancy defects are introduced to the graphene surface. However, to our knowledge, until now few studies have been implemented about the influences of vacancy defects on the GO membrane. So it is important to study the vacancy effects on the wetting properties in GO film.

In this paper, we use a molecular dynamics (MD) simulation method to explore vacancy defect effects on the wetting properties of GO sheets. We elucidate the water density distribution at the interface, as well as the wetting contact angle variation, with a defect concentration density from 0% to 10%. Moreover, the relaxation time (*τ*_c_), the height of the water droplet, and the number of hydrogen bonds on the liquid–solid interface are also investigated.

## 2. Models and Methodology

**Figure 1:**
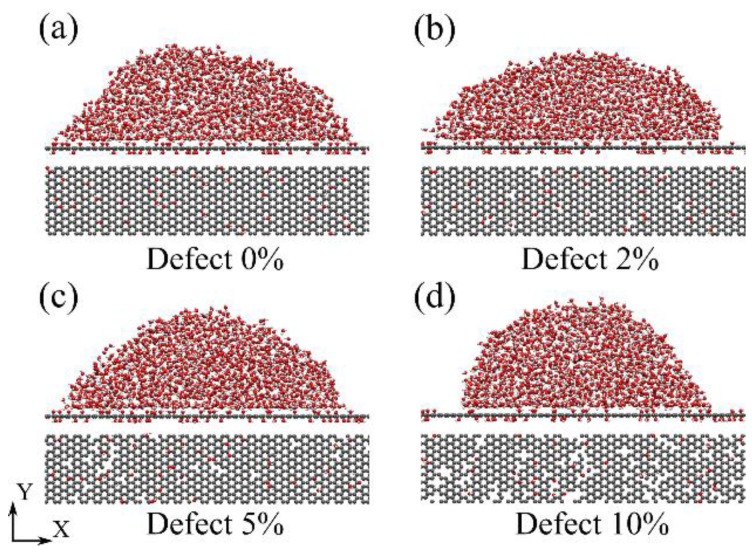
Simulation snapshots of a droplet on the graphene oxide (GO) surface, with various surface vacancy defect concentrations; (**a**–**d**) are the 0%, 2%, 5%, and 10% vacancy defect concentrations, respectively.

Our (MD) simulations were carried out using the large-scale atomic/molecular massively parallel simulator (LAMMPS) [25]. All equilibrium simulations were performed at a constant temperature of 298 K by using the Nosé–Hoover thermostat. The timestep for integrating equations of motion was set to be 1.0 femtosecond (fs). Due to their longevity and greater chemical stability, only hydroxyl groups on the GO surface were considered. The concentration of oxidized groups was defined as *n*_O_/*n*_C_, where *n*_O_ and *n*_C_ are the number of oxygen and carbon atoms in the GO. Here the oxide concentration was fixed at 10%. The vacancy-defective concentration was defined similarly as *n*_MC_*/n*_C_, where *n*_MC_ and *n*_C_ are the removing carbon atom number and the total carbon number of pristine graphene sheet respectively. The oxide groups on GO surface and removing carbon atoms in graphene are setting randomly as shown in Figure 1. Here, the GO was produced by the all-atom optimized potentials for liquid simulations (OPLS-AA), which are able to describe many-body terms related to interatomic interactions, including bond stretching, bond angle bending, dihedral changing, and van der Waals (vdW) and electrostatic interactions [26,27]. The rigid extended simple point charge (SPC/E) model was used for water molecules [28,29]. The SHAKE algorithm was applied to O–H bonds to reduce high frequency vibrations, which requires a smaller timestep for energy conservation of system and numeric integration. The interactions between the GO and water molecules were vdW and electrostatic interactions. The former are described by the 12-6 Lennard Jones (LJ) potential (*V*(*r*_ij_)):

(1) $$V(r_{\mathrm{ij}})=4\varepsilon \left[{\left(\sigma /r_{\mathrm{ij}}\right)}^{12}-{\left(\sigma /r_{\mathrm{ij}}\right)}^{6}\right],$$

where *ε* is the depth of the potential well, *σ* is the zero-crossing distance for the potential, and *r*_ij_ is the distance between the *i* atom and the *j* atom.

**Figure 2:**
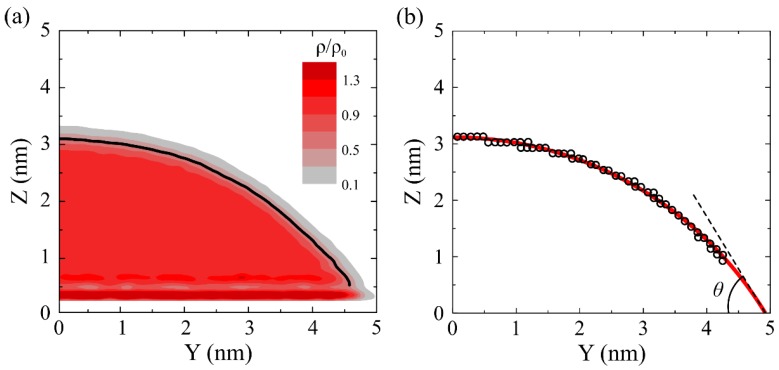
(**a**) Density distribution of water droplet in units of its bulk value. The liquid–vapor interface (the black line) is defined as *ρ*_0_/2. (**b**) A circular best fit (the red line) through these points with a density level at 0.5 g/cm^3^.

The parameters in Equation (1) for oxygen and carbon atom interactions were *ε* = 4.063 meV and *σ* = 0.319 nm, respectively [28]. The cutoff distance of the (LJ) potential was 1.0 nm. The long range Coulomb interactions were computed using the particle–particle particle–mesh (PPPM) algorithm with an accuracy of 0.0001 [30]. In order to eliminate the size effect induced by line tension at the liquid–solid–gas contact line [31], a two dimensional slab model was constructed for the water–GO wetting system. The dimension of graphene oxide was 2.1 nm × 40.0 nm in the *x* and *y* directions. Periodic boundary conditions were applied in these two directions. According to former studies, the width of 2.1 nm is large enough to exclude the size effect in the direction of thickness [12]. Initially, a water droplet with a diameter of 6.0 nm (1500 water molecules) is placed on the top of GO substrate. The wetting contact angle (WCA) of the water droplet is measured when the droplet reaches equilibrium state. The water droplet density distribution was calculated by time-averaging the water density with a mesh grid spacing of 0.05 nm. The density distribution of the droplet is shown in Figure 2a. To obtain the WCA from such a density distribution, a two-step procedure was adopted following the Werder method [32]. First, the boundary of the droplet surface was defined as the contour line with a density level at 0.5 g/cm^3^. Second, a circular best fit through these points was extrapolated to the GO. Then, the WCA was measured, as shown in Figure 2b.

## 3. Results and Discussion

### 3.1. Wettability of Graphene Oxide

**Figure 3:**
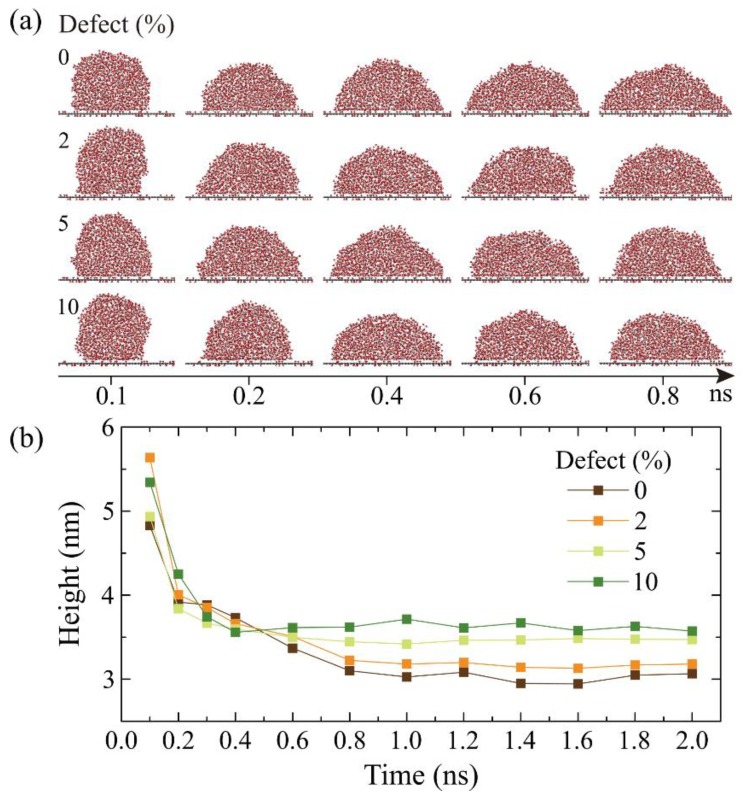
(**a**) Simulation snapshots of a water droplet during the wetting process on different surface vacancy defect concentrations. (**b**) Evolution of the height of the droplet in the simulation.

The spreading behavior of a water droplet and the contact angle (*θ*) are the two key properties to characterize the wettability of GO. First of all, the spreading properties of a water droplet on GO surface is explored, which is important to get the relaxation time *τ_c_* and confirm the time when the system reaches an equilibrium state. A water droplet on pristine graphene can reach the equilibrium state in 0.5 nanosecond(ns), due to the low friction coefficient on the graphene–liquid interface. The relaxation time *τ_c_* increases with increasing oxidized density concentration of the water droplet on the GO. Wei et al. [12] reported that *τ*_c_ is 3.5 ns when the oxidized density concentration is as high as 25%. In order to study the defective concentration (*c*) effects on *τ*_c_, in Figure 3a, we present the snapshots of the water droplet during the spreading process on the graphene oxide surface with different *c*. When the water–GO system approaches its dynamic equilibrium, the *τ*_c_ is up to about 0.8 ns, but the final configurations of the water droplets are nearly the same. Therefore, it is difficult to assess the vacancy defect effects on the spreading properties of GO sheets. Figure 3b shows the height of the water droplet as a function of simulation time *t* on the graphene oxide surface under different values of *c*. The fact that the height of the droplet increases with increasing *c* suggests that the spreading speed on GO sheets decreases with increasing *c*. Moreover, the height of the droplet, which is straight in relation to the contact angle, can be used to present the state of system. The relationship between the height of droplet and contact angle (*θ*) in our present model can be expressed as:

(2) $$h=\sqrt{\frac{\pi r^{2}{\left(1-\cos\theta \right)}^{2}}{\theta -\sin\theta \cos\theta }}$$

where, *h* is the height of water droplet and *r* is the radius of water droplet.

**Figure 4:**
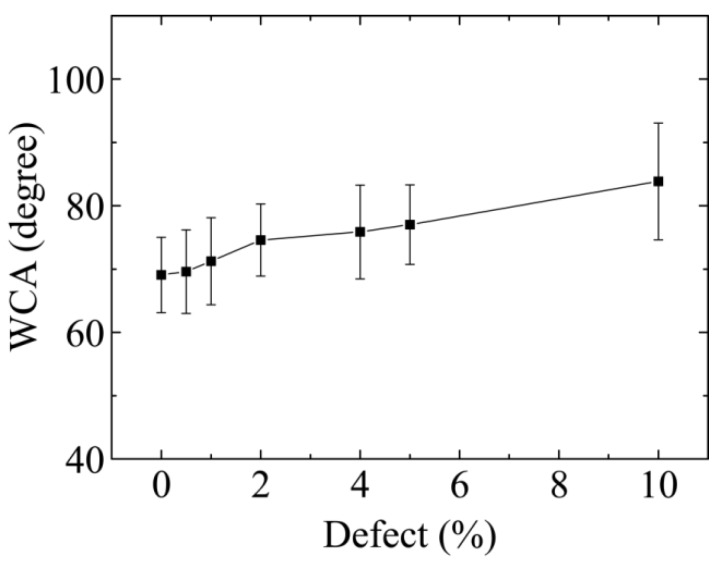
Water contact angles of droplet sessile on GO surface as a function of surface defect concentration.

Then, the WCA of GO with different *c* is computed. To overcome the size limitation for WCA evaluation, we use a two-dimensional (2D) wetting system. This approach has several advantages, such as the elimination of line tension, due to curvature in the Young’s equation compared to that of dome-shaped droplets, and a low requirement for the size of the system to be modeled. In order to verify the present model, we first measured the WCA of a single layer of graphene. The value of WCA on the graphene substrate was 95°, which is consistent with the 91.5° from a modified Young’s equation. WCAs are calculated by averaging the results of five independent simulations with different initial positions of the droplet on the GO surface, since the oxidized groups and defects are distributed randomly on the surface. The relationship between WCA and defect concentration density is shown in Figure 4. The wetting properties of the GO surface decrease with increasing vacancy defects. This tendency is very consistent with previous studies [24]. It means that the vacancies in the GO membrane decrease the interfacial interaction between the surface and water molecules, which results in the weakening of wetting properties. In previous experiments, Walton et al. changed the strength of the interaction between the water and GO by inducing a gradient of oxygen functional groups on the surface of graphene. Thus, the movement of drops of water in the direction of increasing the interaction and the change of wetting properties of GO has been realized [10]. Our results will provide a new method to control the wetting properties of GO, as well as additional capabilities with regard to device design for applications.

### 3.2. Properties of the Water–Graphene Oxide Interface

**Figure 5:**
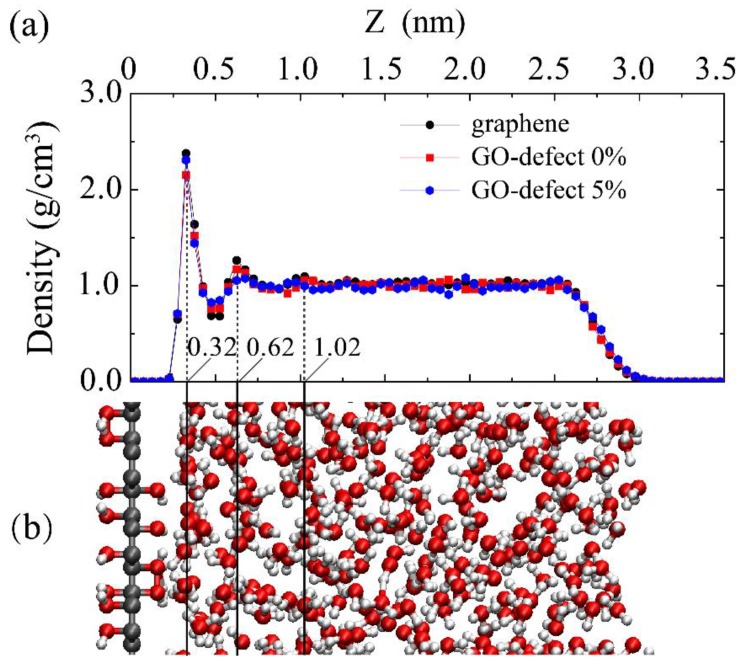
(**a**) Density profile along the GO sheet surface normal direction (*z*-direction). (**b**) Water molecules distributions on GO surface for *c* = 0%.

The interfacial water structures usually determine the wetting properties of water at the interface. Water density profiles, water molecules distributions, and the number of hydrogen bonds at the liquid–solid interface are the basic quantities that characterize the structural features of the interfacial water, in order to study the effects of oxidation and vacancy concentration on the interfacial water density distribution. Density profiles and distributions of the water molecules along the normal surface direction are shown in Figure 5a,b. Results show that the density profiles of water close to the substrate change significantly, due to the presence of the substrate. Three sharp peaks appear in the density curves, indicating that the water near the substrate is much more ordered. Moreover, the three density curves coincide well with each other, especially at the position of the three peaks, which suggests that the vacancy defect and oxidized groups are not able to change the distributions of the water molecules.

**Figure 6:**
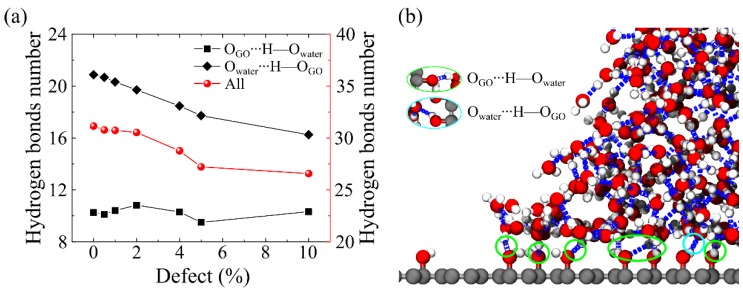
(**a**) Evolution of the number of hydrogen bonds on the liquid–solid interface with defect concentrations. The two black dotted lines represent the number of hydrogen bonds of the donor (bottom line) and acceptor of water molecules (top line). The red dotted line represents the sum of the number of hydrogen bonds between them. (**b**) The schematic diagram of the formation of hydrogen bonds at the liquid–solid contact line. The green circle emphasizes the hydrogen of the donor water molecules, and the cyan circle emphasizes the hydrogen of the acceptor water molecules.

The relationship between the defect concentration and the number of hydrogen bonds at the liquid–solid interface was investigated, in order to illustrate the effect of defect concentrations on the WCA of GO, as shown in Figure 6a. The results suggest that the number of hydrogen bonds between oxidized groups and water molecules decreases with the increasing of *c*, which results in the increasing of the WCA of GO. At the liquid–solid interface, more details of the hydrogen bond can be calculated, including the number of hydrogen bonds of the donor and acceptor water molecules. We can see that the number of hydrogen bonds of the acceptor water molecules is about twice as large as that of the donor water molecules, because there is only one hydrogen atom in the hydroxyl groups. The number of hydrogen bonds of acceptor water molecules have a significant descending trend, while, the number of hydrogen bonds of donor water molecules remain unchanged. Thus, the main factor of the effect of defect concentration on the contact angle is the number of hydrogen bonds of acceptor water molecules. Based on our present results, we can conclude that the increasing vacancy defect concentration in the GO membrane reduces the hydrogen bonds with oxidized groups and water molecules, which in turn leads to an increase in the WCA of GO.

## 4. Conclusions

In present work, the wettability of defective graphene oxide film is studied by molecular dynamics simulations. Our results show that the spreading speed of water on GO sheets decreases with increasing *c*, and the contact angle of the GO increases from 70° to 82° with defect concentrations from 0% to 10%. The vacancy defects in the GO membrane decreases the hydrogen bonds with oxidized groups and water, which in turn leads to an increase in the WCA of GO. Our results provide a new method for controlling the wetting properties of GO, plus additional capabilities for device design for applications.
